# Vegetable Nitrate Intakes Are Associated with Reduced Self-Reported Cardiovascular-Related Complications within a Representative Sample of Middle-Aged Australian Women, Prospectively Followed up for 15 Years

**DOI:** 10.3390/nu11020240

**Published:** 2019-01-22

**Authors:** Jacklyn K. Jackson, Amanda J. Patterson, Lesley K. MacDonald-Wicks, Peta M. Forder, Lauren C. Blekkenhorst, Catherine P. Bondonno, Jonathan M. Hodgson, Natalie C. Ward, Carl Holder, Christopher Oldmeadow, Julie E. Byles, Mark A. McEvoy

**Affiliations:** 1 Faculty of Health and Medicine, School of Health Sciences, Department of Nutrition and Dietetics, University of Newcastle, Callaghan, NSW 2308, Australia; Jacklyn.Jackson@uon.edu.au (J.K.J.); Amanda.Patterson@newcastle.edu.au (A.J.P.); Lesley.Wicks@newcastle.edu.au (L.K.M.-W.); 2 Priority Research Centre for Physical Activity and Nutrition, University of Newcastle, Callaghan, NSW 2308, Australia; 3 Research Centre for Generational Health and Ageing, Hunter Medical Research Institute, University of Newcastle, New Lambton, NSW 2305, Australia; Peta.Forder@newcastle.edu.au (P.M.F.); Julie.Byles@newcastle.edu.au (J.E.B.); 4 School of Medical and Health Sciences, Edith Cowan University, Joondalup, WA 6207, Australia; l.blekkenhorst@ecu.edu.au (L.C.B.); c.bondonno@ecu.edu.au (C.P.B.); jonathan.hodgson@ecu.edu.au (J.M.H.); 5 Medical School, Royal Perth Hospital Unit, University of Western Australia, Perth, WA 6009, Australia; Natalie.Ward@curtin.edu.au; 6 School of Public Health and Curtin Health Innovation Research Institute, Curtin University, Perth, WA 6102, Australia; 7 Clinical Research Design, Information Technology and Statistical Support Unit, Hunter Medical Research Institute, New Lambton, NSW 2305, Australia; Carl.Holder@hmri.org.au (C.H.); Christopher.Oldmeadow@hmri.org.au (C.O.); 8 Faculty of Health and Medicine, School of Medicine and Public Health, Hunter Medical Research Institute, University of Newcastle, New Lambton, NSW 2305, Australia

**Keywords:** dietary nitrate, vegetable nitrate, non-vegetable nitrate, hypertension, heart disease, stroke, thrombosis, cardiovascular disease

## Abstract

Nitric oxide (NO) facilitates anti-atherosclerotic effects. Vegetables are a major source of dietary nitrate. Experimental data indicates that dietary nitrate can significantly reduce major risk factors for atherosclerosis and subsequent cardiovascular disease (CVD), as nitrate can be metabolized to produce NO via the nitrate-nitrite-NO pathway. The purpose of this study was to prospectively investigate the association between habitual dietary nitrate intakes and the incidence of self-reported CVD-related complications within a representative sample of middle-aged Australian women (1946–1951 cohort of the Australian Longitudinal Study on Women’s Health). Women free from disease at baseline who had completed the food frequency questionnaire data were included. Generalized estimating equations were used to estimate odds ratios (OR) and 95% confidence intervals (95% CI) across quartiles for nitrate intakes. Of the 5324 women included for analysis, there were 1951 new cases of CVD-related complications over 15-years of follow-up. Women reporting higher total dietary nitrate intakes (Q4 > 78.2 mg/day) and vegetable nitrate intakes (Q4 > 64.4 mg/day) were 25% and 27% reduced risk of developing CVD-related complications respectively, compared with women reporting low total (Q1 < 45.5 mg/day) and vegetable nitrate intakes (Q1 < 34.8 mg/day). Our findings were consistent with other observational data indicating that dietary nitrate may explain some of the cardiovascular benefits of vegetable consumption.

## 1. Introduction

Cardiovascular disease (CVD) is largely preventable, yet remains the leading cause of death, accounting for half of all non-communicable disease deaths worldwide [1,2]. In Australia, these alarming trends continue. In 2017, CVD accounted for nearly 27% of all deaths in Australia, including 27% of deaths in males and 28% of deaths in females [3]. In addition, CVD is a leading cause of the total burden of disease in Australia, responsible for extremely large social and economic costs, accounting for over 10% of total healthcare expenditure [4]. The prevalence of CVD is expected to rise as the population ages and as rates of overweight and obesity increase, due to continued exposure to major modifiable risk factors, including poor nutrition and low physical activity [5].

It has been estimated that women are 2–3 times more likely to develop hypertension than men, and this represents a major threat to the health of Australian women [6]. In fact, hypertension is frequently cited as one of the most important risk factors for predicting CVD events [7]. Persistent hypertension causes damage to the blood vessels, is responsible for structural changes including the narrowing and stiffening of blood vessels, and contributes to the development of atherosclerosis [8]. On a cellular level, endothelial dysfunction is thought to be a major underlying cause of hypertension [9]. Attenuated nitric oxide (NO) bioavailability is a major characteristic of endothelial dysfunction and is present in arterial hypertension [9]. Low NO bioavailability can occur in states of increased production of reactive oxygen species (ROS) and is associated with poor dietary habits, low physical activity, tobacco use and/or increasing age [9].

In humans, NO is known to have a major role in facilitating vasodilation of the blood vessels and plays an important role in the prevention of platelet adhesion, platelet aggregation and atherosclerosis [10,11]. It has been noted that various pharmacological and non-pharmacological approaches, including regular exercise, good diet and smoking cessation, can improve NO bioavailability and endothelial function in hypertensive populations [12,13,14]. This indicates that low levels of NO bioavailability, subsequent endothelial dysfunction and atherosclerotic effects can be corrected and/or prevented with lifestyle interventions [15].

Dietary optimization is an important lifestyle intervention for the management of existing CVD and its prevention [16]. In particular, there has been a strong emphasis on antioxidant-rich vegetable consumption for CVD prevention, as these are thought to protect against ROS and contain a variety of cardio-protective minerals and phytochemicals, including antioxidants and polyphenols [17].

More recently, it has been postulated that some of the cardiovascular benefits of vegetable consumption could be explained due to the presence of dietary nitrate [15,18]. Rich dietary sources of nitrate include green leafy vegetables and beetroot [19,20]. Dietary nitrate can be metabolized in the human body to produce NO via the enterosalivary nitrate-nitrite-NO pathway, and is thought to be one of the body’s major sources of NO generation in addition to the endogenous L-arginine pathway [18]. Dietary nitrate may be critical for maintaining cardiovascular homeostasis in humans, especially in situations where NO bioavailability (via the L-arginine pathway) is likely compromised, such as chronic disease or aging [21]. This notion is supported by a vast number of published experimental trials indicating that acute intakes of dietary nitrate can significantly improve a variety of CVD risk factors [22]. In a recently published systematic review and meta-analysis of 39 acute experimental trials (≤4 weeks), Jackson et al. reported that intakes of dietary nitrate were significantly associated with a reduction in resting blood pressure, improved endothelial function, reduced arterial stiffness and reduced platelet aggregation [22].

Given the promising results generated from published experimental trials indicating that dietary nitrate can significantly improve major risk factors for atherosclerosis and subsequent CVD, increasing dietary nitrate intakes could represent a possible low cost and simple strategy for decreasing the growing burden of CVD. Currently, there is limited data establishing a relationship between long-term habitual dietary nitrate intake and CVD-related outcomes within large population-based cohorts. Therefore, the aim of the current study was to investigate the association between habitual dietary nitrate intakes (including vegetable derived nitrate and non-vegetable derived nitrate) and incidence of self-reported CVD-related complications including hypertension, heart disease, stroke and thrombosis within a representative sample of middle-aged Australian women prospectively followed up for 15 years.

## 2. Materials and Methods

### 2.1. The Australian Longitudinal Study on Women’s Health (ALSWH)

The ALSWH, also known as the Australian Longitudinal Study on Women’s Health, was established in 1996 to investigate the health and wellbeing of Australian women aged over 20 years. Methods for the ALSWH have been published in detail elsewhere and are available at www.alswh.org.au.

Overall, women in three age groups (1973–1978 cohort, 1946–1951 cohort and 1921–1926 cohort) were randomly selected from the Medicare database (Australia’s government-funded universal healthcare cover) to take part in the first survey conducted in 1996 [23].

Women living in rural and remote areas were intentionally oversampled during recruitment to allow sufficient statistical power to analyze data by area of residence [24]. Based on the original sample recruited in 1996, the ALSWH was a nationally representative sample of over 40,000 women [25].

Ethics approvals for the ALSWH were received from the University of Newcastle (h-076-0795) and the University of Queensland (200400224) and all participants provided informed consent. 

### 2.2. The 1946–1951 Cohort of the ALSWH

Data for this research came only from the 1946–1951 cohort of the ALSWH. This cohort of middle-aged women was surveyed every 2–3 years since the start of the ALSWH in 1996 (women aged 45–50 years) with a total follow-up period of 20 years; the most recent survey was conducted in 2016 (women aged 65–70 years) [25].

Food frequency questionnaire (FFQ) information was collected at two time points, first during Survey 3 in 2001, and again during Survey 7 in 2013. As FFQ information was first collected during Survey 3, 2001 represented the baseline year for this study.

**Figure 1:**
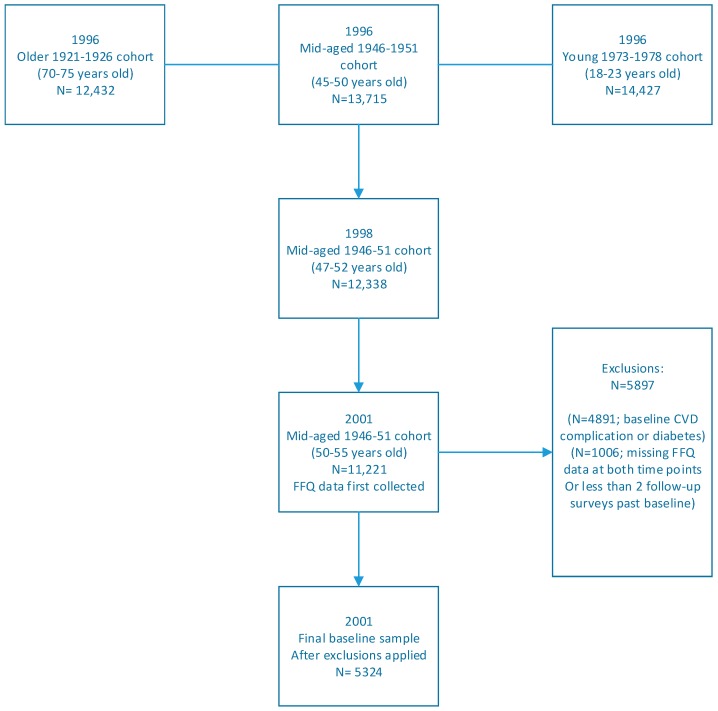
ALSWH: 1946–1951 Cohort Participant Flow Chart.

Women were excluded from analysis if they reported a diagnosis of a CVD-related complication (including hypertension, heart disease, stroke or thrombosis) or diabetes before or at baseline. Women missing FFQ data at either Survey 3 or Survey 7 were excluded. Women were also excluded if they had less than 2 follow-up surveys from baseline (Figure 1).

### 2.3. Assessment of Dietary Intake

Information on dietary intake was obtained from a validated FFQ, known as the Dietary Questionnaire for Epidemiological Studies (DQES) Version 2 [26], which was incorporated as part of Survey 3 and Survey 7 only, collected in 2001 and 2013 respectively from all participants.

The DQES asks participants to report their usual consumption of 74 foods and six alcoholic beverages over the preceding 12 months, using a 10-point frequency option from “never” up to “three or more times per day”. Portion size photographs were used to adjust the serving size for vegetables, meat and casseroles. Additional questions were asked about the total number of daily serves of fruit, vegetables, bread, dairy products, eggs, fat spreads and sugars, as well as the type of bread (wholemeal, wholegrain or white), dairy products (full cream, low fat or skim) and fat spread (butter, monounsaturated, polyunsaturated) used. The development of the DQES and its validation in a sample of Australian women using a 7-day weighted food record have been reported previously [27].

### 2.4. Calculating Total, Vegetable and Non-Vegetable Nitrate Intakes

Dietary nitrate data are not included within National Australian food composition tables, therefore nitrate intakes were estimated based on published nitrate databases.

Vegetable nitrate data were derived from a published database by Blekkenhorst et al. which included worldwide vegetable nitrate data from 255 publications for up to 180 vegetables and 22 herbs and spices [19]. The application of this vegetable nitrate database to 24-hour diet recalls was positively correlated with urinary nitrate (r = 0.4, *p* = 0.013) and its application to the DQES was found to be moderately positively correlated with 24-hour diet recalls (r = 0.5, *p* < 0.001). Vegetable nitrate data from Blekkenhorst et al. were applied to 22 of the DQES items.

Non-vegetable nitrate data were derived from three other key publications, in that nitrate values for 66 DQES items were obtained from Inoue-Choi et al. [28], five were obtained from the Food Standards Australian New Zealand survey of nitrate and nitrite in food and beverages in Australia [29], and nitrate values for two DQES items were obtained from Griesenbeck et al [30].

Responses from the DQES were converted into average daily intakes, and individual food items were calculated in grams per day. The nitrate content of foods was calculated by multiplying the food item in grams by the nitrate content (mg) per gram.

### 2.5. Ascertainment of CVD-Related Complications

CVD-related complications encompassed self-reported doctor diagnoses of hypertension, heart disease, stroke or thrombosis, reported at each survey.

Self-reported data on doctor-diagnosed CVD-related complications were available from each survey. During Survey 1 in 1996, participants were asked if a doctor had ever diagnosed them with CVD related complications. For example, women were asked, “Have you ever been told by a doctor that you have: Heart disease”. At all subsequent surveys, participants were asked if they had been diagnosed with CVD-related complications by a doctor in the last 2–3 years (to coincide with previous surveys). For example, at Survey 2 women were asked, “Have you EVER been told by a doctor that you have hypertension (high blood pressure)?” to which responses included, “Yes, in the last 2 years” and “Yes, more than 2 years ago”. At Survey 3 and subsequent surveys (Surveys 4 to 8), women were asked “In the PAST THREE YEARS, have you been diagnosed or treated for: Heart disease (including heart attack, angina), hypertension (high blood pressure), stroke or thrombosis (blood clot)”. The question about thrombosis was asked during all survey periods, except Survey 5. 

If women reported having hypertension, heart disease, stroke or thrombosis during Surveys 1 to 3, they were excluded from the analysis. Incidence of CVD-related complications was defined as a new report of hypertension, heart disease, stroke or thrombosis at Surveys 4 to 8. Previously, the examination between self-reported hypertension and hypertension medication use in the 1946–1951 cohort indicated a high level of agreement between measures (89%) [31].

However, due to the possibility that CVD-related events had been misreported, we also conducted a sensitivity analysis. As part of this sensitivity analysis, CVD-related complications were only considered if the outcomes were reported in two or more follow-up surveys. The exception to this was if a CVD-related complication was reported for the first time at the most recent survey (Survey 8, collected in 2016).

### 2.6. Covariates

At every survey, participants were asked to provide information on a range of demographic and socio-economic factors and health risk behaviors. Covariates relevant to the current analysis included socio-economic status (SES), level of education, smoking status, body mass index (BMI), physical activity levels, country of birth, level of alcohol intake and menopause status. SES was determined based on how well participants reported they could manage on the income they had available. Response options were categorized as: Low, “It is impossible” or “It is difficult all the time”; intermediate, “It is difficult some of the time” or “It is not too bad”; and high, “It is easy” [32]. Level of education was determined as: Low, if the highest level of education reported was “No formal qualifications” or “School or intermediate certificate or equivalent”; intermediate if the response was “High school or leaving certificate, trade/apprenticeship, or certificate or diploma”; and high, if the level of education was reported as “University degree or postgraduate degree”. Smoking status was defined as “Non-smoker”, “Former smoker” or “Current smoker”. 

Participants were asked to report their height and weight, and from this their body mass index (BMI) was calculated and categorized as either underweight (BMI < 20 kg/m^2^), healthy weight (BMI 20–25 kg/m^2^), overweight (BMI > 25–30 kg/m^2^) or obese (BMI > 30 kg/m^2^) [33]. Self-reported BMI data from ALSWH were previously validated [34]. Physical activity levels were derived from validated questions on the frequency and duration of walking (for recreation or transport) and from moderate- and vigorous intensity activity in the last week [35]. From this, physical activity levels were defined as “Sedentary/low” (<600 total metabolic equivalents (MET (minutes/week)) or “Moderate/high” (≥600 MET minutes/week) [36]. At Survey 1, participants were asked to report their country of birth, and responses were categorized as “Australian born”, “Other English Speaking Background”, “Europe”, “Asia” and “Other”. Alcohol status was also derived and categorized as per National Health and Medical Research Council (NHMRC) alcohol guidelines. Categories included “Low risk drinker”, “Non-drinker”, “Rarely drinks”, “Risky drinker”, and “High risk drinker” [37]. Menopause status was determined using questions on hysterectomy, oophorectomy, hormone therapy and menstrual patterns, and categorized as “hysterectomy and/or oophorectomy”, “hormone therapy use”, “pre-menopausal”, “peri-menopausal” or “post-menopausal” [38].

### 2.7. Covariate Selection

Using the software program DAGitty [39], a Directed Acyclic Graph (DAG) was used to determine confounders of the association between dietary nitrate and self-reported CVD-related complications, based on the literature indicating multiple potential confounding variables (Supplementary Figure S1). From the causal diagram shown in Supplementary Figure S1, three potential confounders were identified. These included SES, education and dietary source of nitrate. Upon further analysis we found statistically significant associations between each potential confounder with both dietary nitrate intakes (exposure) and self-reported CVD-related complications (outcome), in accordance with the definition of confounding variables [40]. However, the DAG also indicated that controlling for just the major dietary source of nitrate intake would be the minimal sufficient adjustment required for estimating the total effect of dietary nitrate on self-reported CVD-related complications. Therefore, we present three different models. Model 1 is adjusted for SES and education (non-dietary confounders), Model 2 is adjusted for Model 1 as well as the major dietary source of nitrate intake (e.g., vegetable intake in grams/day) (non-dietary and dietary confounders), and Model 3 is adjusted for Model 2 variables plus energy (kJ/day).

### 2.8. Analysis

We analyzed the prospective association between total nitrate, vegetable nitrate and non-vegetable nitrate intakes (based on FFQ data collected at Survey 3 (used for Surveys 3 to 6), and updated at Survey 7 (used for Surveys 7 and 8)) and incidence of combined self-reported CVD-related complications including hypertension, stroke, heart disease and thrombosis (Surveys 4 to 8), using Generalized Estimating Equations (GEE). An independent correlation structure was used for the GEE model, due to the 12-year gap between individual nitrate measurements. This structure was supported by an improved model fit, as evidenced by a lower QIC value. Each participant contributed only one endpoint and the cohort at risk of each 3-year follow-up period included only those who had not yet reported a CVD-related event, at the beginning of each follow-up period. If women had missing FFQ data at either Surveys 3 or 7, they were excluded from the analysis. 

For analyses, participants were divided into quartiles based on their level of total nitrate, vegetable nitrate and non-vegetable nitrate consumption. The lowest quartile for nitrate intakes represented the reference category. 

A sensitivity analysis was conducted based on CVD case definition, as described previously. We also conducted a sensitivity analysis, including only hypertension cases, given that hypertension accounts for the majority of cases, and there was convincing experimental data indicating that dietary nitrate intakes could exert blood pressure lowering effects.

GEE models with time-varying covariates were used for all analyses using STATA 14.2 (StatCorp, College Station, TX, USA), to estimate Odds Ratios (ORs) and corresponding 95% confidence intervals (CIs). Statistical significance was defined as *p* < 0.05.

## 3. Results

### 3.1. Characteristics of Study Population 

A total of 5324 women were included at baseline (2001) (Figure 1), with a mean age of 52.4 years (SD: 1.5 years). Women with higher intakes of total dietary nitrate were significantly more likely to have a higher BMI, be Australian-born and use a multi-vitamin or other supplement. Women reporting higher intakes of total dietary nitrate were also less likely to live in urban areas, less likely to smoke or partake in risky or high risk alcoholic drinking behaviors (Table 1).

**Figure 2:**
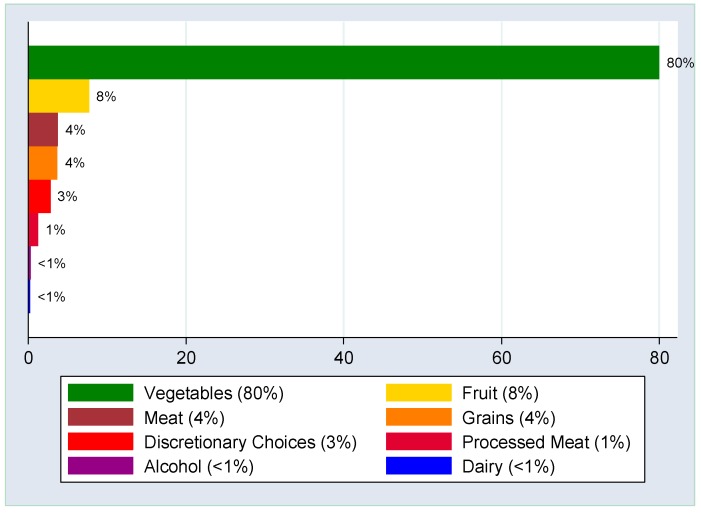
Percent contribution to total dietary nitrate intakes by food group. Vegetables contributed approximately 80% of total nitrate intakes, followed by fruit (8%), meat (4%), grains (4%), discretionary choices (3%), and processed meat (1%). Alcohol and dairy contributed very minor amounts of nitrate.

The median total nitrate intake for our sample was 60.3 mg/day (IQR: 32.7 mg/day), and the median vegetable nitrate intake was 47.9 mg/day (IQR: 27.6 mg/day). On average vegetable nitrate intakes accounted for approximately 80% of total nitrate intakes. Other major sources of dietary nitrate included fruit (8%), meat (including red meat and poultry) (4%), grains (4%), discretionary choices (including take-away foods, pastries and confectionary) (3%) and processed meats (1%) (Figure 2).

Women reporting higher intakes of total dietary nitrate were significantly more likely to report higher dietary intakes of many dietary components, including vegetable serves, fruit serves and meat serves. Women reporting higher intakes were significantly more likely to report a higher total percent energy contributed from protein and a lower total percent energy contributed from fat, but the total percent energy contributed from carbohydrate did not differ across quartiles for total nitrate intakes (Table 2).

### 3.2. Incidence of Self-Reported CVD Related Complications

During 15 years of follow-up, 1951 of 5324 participants (36.6%) reported a CVD-related complication (Table 3). In total, approximately 30% of participants reported a diagnosis of hypertension, 8.5% reported a diagnosis of heart disease, 3% reported a diagnosis of thrombosis and 2% reported a diagnosis of stroke.

The relationship between quartiles of total nitrate intakes and self-reported CVD-related events are reported in Table 3. Women reporting higher total dietary nitrate intakes (Q4 > 78.2 mg/day) were at a 25% (OR: 0.75 (95% CI: 0.63–0.91); *p* for trend = 0.02) lower risk of self-reported CVD-related events, compared with women reporting low total nitrate intakes (Q1 < 45.5 mg/day) in our multivariate model (model 3).

A statistically significant inverse association was observed across quartiles for vegetable nitrate intakes and self-reported CVD-related events (Table 4). In our multivariate model, women reporting higher intakes of vegetable derived nitrate (Q4 > 64.4 mg/day) were observed to be at a 27% (OR: 0.73 (95% CI: 0.61–0.88); *p* for trend = 0.01) lower risk of self-reported CVD-related complications, compared with women consuming low vegetable derived nitrate (Q1 < 34.8 mg/day). 

When women were ranked based on their intakes of non-vegetable derived nitrate there was no statistically significant association with self-reported CVD-related complications (Table A1). When non-vegetable nitrate intakes were grouped by dietary source, we observed a trend for the increasing risk of CVD-related complications with increasing intakes of non-vegetable derived nitrate from meat, processed meat, discretionary choices and alcohol. Although in our fully adjusted model, the trend across intakes were not statistically significant, there was a statistically significant increased risk of CVD when comparing highest intakes (Q4: >8.1mg/day) to lowest intakes (Q1: <3.6 mg/day) (OR: 1.27 (95% CI: 1.07–1.51), *p* < 0.05).

In addition, we observed an inverse association between CVD-related complications and intakes of non-vegetable derived nitrate from fruit, grains and dairy, however in our fully-adjusted model this relationship was not statistically significant (OR: 0.88 (95% CI: 0.70–1.09); *p* for trend = 0.6).

### 3.3. Sensitivity Analyses

We applied a strict criterion for defining our self-reported CVD-related complications as part of our sensitivity analyses, in which we only counted CVD-related events if reported at more than one follow-up survey, or if events were reported for the first time during Survey 8. Results for these analyses are presented in Table A2. Applying a more conservative case definition, 1586 out of 5324 (29.7%) participants reported cases of CVD-related complications. Based on this, we no longer observed a statistically significant trend for total dietary nitrate intakes and CVD-related complications, however there was a statistically significant lower risk of CVD-related complications for women grouped in Quartile 4, compared with Quartile 1 (OR: 0.78 (95% CI: 0.63–0.95); *p* < 0.05). A statistically significant inverse association was maintained across quartiles for vegetable nitrate intakes, as higher intakes were associated with a 25% (OR: 0.75 (95% CI: 0.61–0.91); *p* for trend = 0.04) lower risk of CVD-related complications. For non-vegetable derived nitrate intakes, our findings did not change substantially from the main analysis findings. 

During 15 years of follow-up, 1615 participants reported having hypertension. The relationship between total nitrate intakes and self-reported hypertension are reported in Table A3. Although our fully adjusted model for total nitrate intakes did not detect a statistically significant trend for lowered hypertension risk (*p* for trend = 0.06), there was a statistically significant lower risk of hypertension for women grouped in Quartile 4 for total nitrate intakes, compared with Quartile 1 (OR: 0.78 (95% CI: 0.63–0.95), *p* < 0.05). On the other hand, when this association was analyzed based on quartiles of total vegetable nitrate intakes, a statistically significant lower risk of hypertension was detected across increasing vegetable nitrate quartiles, with the lowest risk detected in participants reporting nitrate intakes in Quartile 4 (OR: 0.74 (0.61–0.90), *p* for trend = 0.02).

## 4. Discussion

Among middle-aged Australian women, we found statistically significant inverse associations between total dietary nitrate intakes and vegetable nitrate intakes and incidences of self-reported CVD-related complications over a 15-year follow-up period. However, it was interesting to note, that the source of dietary nitrate largely determined both the direction of effect and the size of the effect.

On average, women within our sample were consuming approximately 61 mg/day total nitrate and 48 mg/day vegetable nitrate at baseline. This level of nitrate intake was markedly lower than mean nitrate intakes estimated in previous Australian based cohort studies, including the Calcium Intake Fracture Outcome Study (CAIFOS) (total nitrate ~79 mg/day; vegetable nitrate ~67mg/day) [41] and the Blue Mountains Eye Study cohort (BMES) (total nitrate ~129 mg/day; vegetable nitrate ~110 mg/day) [42]. These large differences in estimated nitrate intakes have occurred despite using the same nitrate databases to estimate intakes [19,28,29,30]. On the other hand, it is likely these differences in estimated nitrate intakes could largely be explained due to key differences in the cohort populations, and the dietary assessment tool used. For example, both the present study and CAIFOS applied the same nitrate databases to the same FFQ (DQES), however a key difference between the cohort participants was their age. In the current study, we used FFQ data collected at two time points, in 2001 when the women were 50–55 years old, and in 2013 when women were 62–67 years old. This 12-year observation period and age increase represents a period of time in which significant lifestyle changes are likely to occur in women, and consistent with our previous investigations, dietary nitrate intakes appear to increase with increasing age [20]. Based on the FFQ data collected in 2013, the mean total nitrate intake was 72 mg/day and mean vegetable nitrate intake was 60 mg/day. This level of intake was still lower than nitrate intakes estimated in the CAIFOS, which collected FFQ data from women aged 70–85 years old in 1998 [41]. We previously theorized that this difference could be explained by the significant changes in the Australian food environment in recent years, in which case it is likely the DQES was able to capture whole dietary intakes in 1998 better, compared to dietary intakes in 2013 [20]. On the other hand, we expect there are two major reasons why our cohort has lower estimated nitrate intakes than those reported by the BMES, including differences in the FFQ tool and cohort population. Unlike the ALSWH and CAIFOS cohorts which use the 74 item DQES and include only female participants, the BMES includes female and male participants aged >45 years old, and assessed diet using a 145 item FFQ [42]. This is an important factor to consider in this context, given men and women differ in their preferences for food items and consumption amounts, thus it is reasonable to expect that gender differences could be driving these inconsistencies [43]. 

Despite the differences in estimated nitrate intakes across the different cohorts, the percentage contribution of nitrate from food groups remained consistent, with combined vegetable and fruit nitrate intakes accounting for approximately 90% of total nitrate intakes in all cohorts. With this in mind, it is increasingly apparent that the source of dietary nitrate could play a vital role in the long-term physiological effects nitrate may have on the body, and likely explains why we previously found that total nitrate intakes were significantly associated with improved diet quality scores [20]. 

Both Blekkenhorst et al. [41] and Liu et al. [42] recently reported that the association for intakes of total nitrate were similar to vegetable nitrate, but non-vegetable nitrate was not associated with CVD mortality within the CAIFOS and BMES cohorts, respectively. Our findings were consistent with this literature, in that total nitrate intakes and vegetable nitrate intakes were observed to have a significant inverse association with self-reported CVD-related complications including hypertension, heart disease, stroke and thrombosis, observing a combined reduction in risk of 25% and 27% respectively with higher intakes. However, we suspect that as vegetable nitrate accounted for such a large portion of total nitrate intakes, vegetable nitrate was largely driving this association, given that total non-vegetable nitrate intakes were not associated with self-reported CVD-related complications (Q4; OR: 1.10 (95% CI: 0.91–1.33); *p*-trend = 0.3). Yet, when we analyzed non-vegetable nitrate intakes based on dietary source, a statistically significant association emerged, but unlike vegetable nitrate which was associated with a reduction in self-reported CVD-related complications, non-vegetable nitrate from meat, processed meat, discretionary choices and alcohol were associated with an increased risk of CVD-related complications (Q4; OR: 1.27 (95% CI: 1.07–1.51); *p*-trend = 0.06).

These findings are possibly not surprising, and on a metabolic level this paradoxical effect makes sense. In fact, research surrounding dietary nitrate historically focused on intakes from processed meat and their association with poor health outcomes, primarily cancer [44]. In addition, it is reasonable to expect that not all sources of nitrate are equal with regards to their potential health effects [45]. This is especially relevant given the complex bioactivity of nitrate, nitrite and NO metabolism, in which pro-inflammatory constituents including sodium and saturated fat intakes (common to processed meat and discretionary choices) can interfere with NO production, while anti-inflammatory constituents including vitamin C, polyphenols and antioxidants (common to vegetables) can favor NO production from nitrate [46,47,48]. However, understanding how dietary nitrate is metabolized within the food matrix remains a specialized area of research which requires further investigation.

Unlike the studies by Blekkenhorst et al. [41] and Liu et al. [42] which examined the association between dietary nitrate intakes and CVD mortality using linked mortality data, the outcome for the current investigation relied on self-reported doctor diagnoses of either hypertension, heart disease, stroke or thrombosis, which represented a possible limitation of our study. It is important to recognize that use of self-reported CVD-related events is representative of non-fatal CVD events only (rather than total i.e., non-fatal and fatal). Thus, given women tend to experience relatively high rates of fatal CVD events, there is a good chance we have underestimated the risk of total cardiac events. Based on our case definition using self-reported data in our cohort, however, self-reported CVD related events are consistent with those expected within a female Australian population. For example, data from the Australian Bureau of Statistics estimates that about 32.3% of Australian adult females have hypertension, and in our cohort about 30% of participants reported having a diagnosis of hypertension [49]. Coronary heart disease (CHD), including heart attack and angina, is thought to affect 4% of Australian women aged 55–59 years, and peak at 20% among those aged 85 years and older. In our cohort we followed women aged 50–55 years until they were 62–67 years, and found 8.5% of study participants reported a diagnosis of heart disease [50]. Stroke is estimated to occur in 2% of Australian women, which matches the prevalence of stroke reported by our cohort, and use of self-related stroke data was previously validated in this cohort [50,51]. In addition, Ageno et al. found that the cumulative probability of having a thromboembolic event was 0.5% at 50 years, 2% at 60 years, and 8.2% at 75 years, which also approximately matched the prevalence of thrombosis reported in our cohort [52]. Despite our data matching wider incidence data for CVD-related complications, however, we conducted a sensitivity analysis, in which cases of self-reported CVD-related complications were defined based on a more conservative definition. Based on our sensitivity analysis the relationship for total nitrate intakes was attenuated, however a significant inverse association for vegetable nitrate intakes and CVD-related complications remained. These findings reinforced our overall message, that vegetable nitrate sources could be a key factor to promote for CVD prevention. Although nitrate-rich green leafy vegetables are already promoted to prevent CVD, these findings add to the growing evidence that in addition to components of green leafy vegetables including potassium and vitamin K, nitrate may also play an important role. 

In saying that, experimental data using high nitrate diets (rich in green leafy vegetables), have not provided convincing results, opposed to studies which have used beetroot juice [22]. This point was highlighted in a recent meta-analysis, which showed that beetroot juice intakes were associated with significant reductions in resting blood pressure (systolic: −5.7 mmHg (*p* < 0.0001); diastolic: −2.4 mmHg (*p* < 0.0001)), but high nitrate diets were not (systolic: −2.4 mmHg (*p* = 0.2); diastolic: −0.6 mmHg (*p* = 0.5)) [22]. Previously, the major issue with high nitrate diet interventions was thought to be due to the possible high variability in nitrate contents of green leafy vegetables, which can vary considerably depending on cultivation conditions, farming practices, cooking and processing [19,53,54]. This represents a major limitation of this field of research, impeding our ability to accurately estimate nitrate intakes. In addition, it is possible that given the short time frame of these high nitrate diet trials (1–10 days), the external validity of the study findings were compromised, as it is unrealistic that any dietary alterations for a few days could have lasting long-term clinical implications. It is therefore interesting to note that currently very few observational studies have prospectively investigated the relationship between dietary nitrate intakes and hypertension. Results from our analysis found that women consuming vegetable nitrate in the highest quartile (>64.4 mg/day) were at a 26% lower risk of reporting hypertension compared to women reporting vegetable nitrate in the lowest quartile (<34.8 mg/day). This is consistent with findings reported by Golzarland et al. who observed a significant inverse association between nitrate containing vegetable consumption and the 3-year risk of hypertension (~439 g/day: OR: 0.63 (95% CI: 0.41–0.98), *p* = 0.05) in adults aged 20–70 years old [55]. Given the public health challenges faced by the increasing prevalence of hypertension across the globe, increasing the emphasis on the consumption of nitrate-rich vegetables may have important public health implications.

A limitation of our study is the observational nature, limiting our ability to confirm a causal relationship, as we cannot exclude the possibility that findings are the result of residual confounding. As previously mentioned, however, there is strong evidence from clinical trials demonstrating a relationship between nitrate intake and vascular health, supporting potential benefits of nitrate intake on cardiovascular health. It is also worth noting that we have controlled for the major confounders related to this relationship, using evidenced based methodology. In addition, long-term prospective cohort studies are the strongest observational study design, as their prospective nature makes them less prone to biases, including recall and selection biases common to retrospective or cross-sectional studies.

## 5. Conclusions

Our study demonstrated an inverse relationship between vegetable nitrate intakes and the incidence of self-reported CVD-related complications, including hypertension, heart disease, stroke and thrombosis within a large representative sample of Australian middle-aged women, followed-up over 15 years. Our findings are in line with other Australian based studies, indicating that an improved long-term habitual intakes of vegetable nitrate may represent a possible public health strategy for reducing the burden of CVD-related events. Future studies are required to help build an understanding of how dietary nitrate as part of the food system and whole dietary intakes influences health outcomes of the population.

## Figures and Tables

**Table 1:** Baseline characteristics of all study participants by quartile for total dietary nitrate intakes.

	Q1 (<45.5 mg/day)	Q2 (45.5–60.2 mg/day)	Q3 (60.3–78.2 mg/day)	Q4 (>78.2 mg/day)	*p*-Value for Trend
Women (*n*)	1331	1331	1331	1331	
Age (years)	52.4 ± 1.5	52.4 ± 1.5	52.4 ± 1.5	52.4 ± 1.4	0.96
BMI (kg/m^2^)	24.5 ± 5.3	24.8 ± 5.6	25.1 ± 5.7	25.0 ± 5.6	0.003
Australian born (%)	70.4%	77.2%	79.5%	80.7%	<0.0001
Urban area of residence (%)	41.6%	38.2%	34.1%	29.7%	<0.0001
Highly educated (%)	19.7%	17.8%	17.2%	17.0%	0.09
Overweight or obese (%)	44.2%	46.7%	50.6%	49.5%	0.02
Smoking (%)					
Current	14.9%	13.6%	11.5%	12.2%	0.007
Former	22.8%	23.8%	24.8%	26.5%	
Risky/High risk alcohol drinker (%)	4.3%	6.2%	5.4%	4.8%	0.03
Physically inactive (%)	57.0%	53.0%	50.0%	47.0%	<0.0001
Post-menopausal (%)	26.0%	25.4%	24.5%	25.6%	0.01
Hormone replacement therapy use (%)	32.0%	31.0%	30.8%	30.9%	0.89

Total baseline sample = 5324. Quartiles based on total dietary nitrate intakes. Age is mean ± standard deviation (sd). BMI is medians ± IQR. Not complete observations for: BMI (*n* = 5039), country of birth (*n* = 5278), smoking status (*n* = 5312), menopausal status (*n* = 5296), physical activity level (*n* = 5140), BMI category (*n* = 5,039), area of residence (*n* = 5303), level of education (*n* = 5290), and NHMRC alcohol status (*n* = 5038). *p*-value represents level of significance for difference found through ANOVA for continuous variables and Chi-2 tests for categorical variables.

**Table 2:** Summary of dietary intakes for all study participants by quartile for total dietary nitrate intakes.

	Q1 (<45.5 mg/day)	Q2 (45.5–60.2 mg/day)	Q3 (60.3–78.2 mg/day)	Q4 (>78.2 mg/day)	*p*-Value for Trend
Total nitrate intake (mg/day)	36.2 ± 11.9 5	53.2 ± 7.3	68.1 ± 8.8	94.2 ± 23.6	<0.0001
Vegetable nitrate intake (mg/day)	26.4 ± 11.3	41.9 ± 7.4	55.1 ± 9.1	79.3 ± 22.3	<0.0001
Vegetable serves/day	1.3 ± 0.7	1.9 ± 0.7	2.3 ± 0.8	3.1 ± 1.2	<0.0001
Fruit serves/day	1.3 ± 1.2	1.7 ± 1.4	1.9 ± 1.5	2.5 ± 1.7	<0.0001
Grain serves/day	2.9 ± 1.9	3.2 ± 1.9	3.5 ± 2.0	3.8 ± 2.4	<0.0001
Dairy serves/day	1.6 ± 0.9	1.7 ± 1.0	1.7 ± 0.9	1.8 ± 1.0	<0.0001
Processed meat serves/day	0.2 ± 0.3	0.2 ± 0.3	0.2 ± 0.3	0.2 ± 0.4	<0.0001
Meat serves/day	1.3 ± 0.9	1.5 ± 1.0	1.8 ± 1.2	2.2 ± 1.5	<0.0001
Discretionary choices serves/day	1.4 ± 1.3	1.6 ± 1.4	1.7 ± 1.5	1.9 ± 1.8	<0.0001
Energy, kJ/day	5327 ± 2257	6048 ± 2292	6565 ± 2663	7333 ± 3070	<0.0001
Total fat, g/day	50.8 ± 25.8	56.1 ± 28.5	60.3 ± 31.3	66.4 ± 37.0	<0.0001
Total energy contribution from fat, %	36.4 ± 8.0	35.8 ± 8.0	35.2 ± 8.0	34.5 ± 7.9	<0.0001
Saturated fat, g/day	20.1 ± 12.4	21.8 ± 12.8	23.5 ± 14.2	25.7 ± 16.1	<0.0001
Monounsaturated fat, g/day	17.3 ± 9.1	19.8 ± 10.2	21.4 ± 11.5	23.8 ± 14.0	<0.0001
Polyunsaturated fat, g/day	7.6 ± 5.7	8.5 ± 6.0	9.4 ± 6.6	10.3 ± 7.0	<0.0001
Dietary cholesterol, mg/day	184.3 ± 93.8	206.7 ± 100.4	226.5 ± 122.5	255.2 ± 141.0	<0.0001
Protein, g/day	63.3 ± 26.5	71.1 ± 26.8	79.7 ± 33.0	91.6 ± 39.0	<0.0001
Total energy contribution from protein, %	19.6 ± 4.0	20.3 ± 3.9	20.3 ± 3.8	21.0 ± 4.4	<0.0001
Carbohydrate, g/day	140.0 ± 62.3	158.8 ± 64.4	172.8 ± 69.0	197.7 ± 85.1	<0.0001
Total energy contribution from carbohydrate, %	44.5 ± 7.4	44.5 ± 8.0	44.8 ± 7.7	45.0 ± 8.2	0.4
Sugar, g/day	65.3 ± 32.5	73.6 ± 33.7	80.0 ± 37.8	92.2 ± 44.9	<0.0001
Fibre, g/day	14.4 ± 7.1	17.5 ± 7.3	20.3 ± 7.8	24.4 ± 11.0	<0.0001
Alcohol, g/day	3.9 ± 12.9	4.8 ± 15.8	5.6 ± 15.7	4.2 ± 15.3	0.01
Sodium, mg/day	1672.0 ± 780.2	1883.3 ± 781.8	2048.3 ± 908.7	2301.7 ± 1109.7	<0.0001
Potassium, mg/day	1999.3 ± 754.3	2353.1 ± 778.9	2653.9 ± 897.6	3149.8 ± 1143.0	<0.0001
Magnesium, mg/day	203.3 ± 87.8	234.8 ± 91.0	263.5 ± 100.9	311.0 ± 129.0	<0.0001
Vitamin C, mg/day	71.3 ± 54.8	94.8 ± 61.2	109.8 ± 71.7	142.5 ± 85.2	<0.0001

Quartiles based on level of total nitrate intake. Values are medians and inter-quartile ranges; this is to account for possible extreme values (no exclusions have been made based on extreme dietary intakes). *p*-value represents level of significance for difference found through ANOVA for continuous variables.

**Table 3:** Odds of self-reported CVD-related complications by quartile of total nitrate intake in the 1946–51 cohort of the ALSWH (2001–2016) *.

	Q1 (<45.5 mg/day)	Q2 (45.5–60.2 mg/day)	Q3 (60.3–78.2 mg/day)	Q4 (>78.2 mg/day)	
Number of self-reported CVD related cases	505	504	495	447	
	**Q1** **OR (95% CI)**	**Q2** **OR (95% CI)**	**Q3** **OR (95% CI)**	**Q4** **OR (95% CI)**	***p*-Value for Trend**
Un-adjusted Model	1 [Reference]	0.98 (0.86–1.11)	0.95 (0.84–1.09)	0.86 (0.76–0.98) ^a^	0.1
Model 1	1 [Reference]	0.98 (0.87–1.12)	0.95 (0.83–1.08)	0.86 (0.75–0.98) ^a^	0.1
Model 2	1 [Reference]	0.95 (0.83–1.09)	0.89 (0.77–1.03)	0.77 (0.64–0.92) ^a^	0.03
Model 3	1 [Reference]	0.95 (0.83–1.08)	0.88 (0.76–1.02)	0.75 (0.63–0.91) ^a^	0.02

Abbreviations: CVD: Cardiovascular disease; ALSWH: Australian Longitudinal Study on Women’s Health; OR: Odds Ratio; 95% CI: 95% Confidence Interval. * Total nitrate intake based on Survey 3 FFQ data from baseline (2001) until Survey 6, and updated at Surveys 7 and 8 based on Survey 7 FFQ data (2013). Model 1: adjusted for non-dietary confounders including SES and education. Model 2: Model 1 + dietary confounders including vegetable consumption (grams/day). Model 3: Model 2 + energy (kJ/day). a: Indicates OR is statistically significantly (*p* < 0.05) difference from Q1.

**Table 4:** Odds of self-reported CVD-related complications by quartile of vegetable nitrate intake in the 1946–1951 cohort of the ALSWH (2001–2016).

	Q1 (<34.8 mg/day)	Q2 (34.8–47.8 mg/day)	Q3 (47.9–64.4 mg/day)	Q4 (>64.4 mg/day)	
Number of self-reported CVD related cases	523	493	488	447	
	**Q1** **OR (95% CI)**	**Q2** **OR (95% CI)**	**Q3** **OR (95% CI)**	**Q4** **OR (95% CI)**	***p*-Value for Trend**
Un-adjusted Model	1 [Reference]	0.92 (0.91–1.05)	0.90 (0.79–1.03)	0.83 (0.73–0.95) ^a^	0.053
Model 1	1 [Reference]	0.92 (0.81–1.05)	0.90 (0.79–1.03)	0.83 (0.73–0.95) ^a^	0.05
Model 2	1 [Reference]	0.89 (0.78–1.02)	0.84 (0.73–0.98) ^a^	0.73 (0.61–0.88) ^a^	0.009
Model 3	1 [Reference]	0.89 (0.78–1.02)	0.85 (0.73–0.98) ^a^	0.73 (0.61–0.88) ^a^	0.01

Abbreviations: CVD: Cardiovascular disease; ALSWH: Australian Longitudinal Study on Women’s Health; OR: Odds ration; 95% CI: 95% Confidence Interval. Model 1: adjusted for non-dietary confounders including SES and education. Model 2: Model 1 + dietary confounders including vegetable consumption (grams/day). Model 3: Model 2 + energy (kJ/day). a: Indicates OR is statistically significantly (*p* < 0.05) difference from Q1.

## Supplementary Materials

The following are available online at http://www.mdpi.com/2072-6643/11/2/240/s1, Figure S1: Directed Acyclic Graph (DAG), used to identify confounders between dietary nitrate and self-reported cardiovascular disease.

## Appendix A

**Table A1:** Odds of self-reported CVD related complications by quartile of non-vegetable nitrate intakes in the 1946–1951 cohort of the ALSWH (2001–2016).

Total Non-Vegetable Nitrate Intakes	Q1 (<8.9 mg/day)	Q2 (8.9–11.7 mg/day)	Q3 (11.8–14.9 mg/day)	Q4 (>14.9 mg/day)	
Number of self-reported CVD related cases	497	460	485	509	
**Total Non-Vegetable Nitrate Intakes ***	**Q1** **OR (95% CI)**	**Q2** **OR (95% CI)**	**Q3** **OR (95% CI)**	**Q4** **OR (95% CI)**	***p*-value for trend**
Un-adjusted Model	1 [Reference]	0.90 (0.79–1.03)	0.97 (0.85–1.10)	1.02 (0.89–1.16)	0.3
Model 1	1 [Reference]	0.91 (0.80–1.04)	0.97 (0.85–1.11)	1.01 (0.89–1.15)	0.4
Model 2	1 [Reference]	0.94 (0.82–1.08)	1.03 (0.89–1.20)	1.08 (0.90–1.31)	0.4
Model 3	1 [Reference]	0.95 (0.83–1.09)	1.05 (0.90–1.22)	1.10 (0.91–1.33)	0.3
**Non-vegetable nitrate from meat etc.**	**Q1** **(<3.6 mg/day)**	**Q2** **(3.6-5.5 mg/day)**	**Q3** **(5.6-8.1 mg/day)**	**Q4** **(>8.1 mg/day)**	
Number of self-reported CVD related cases	428	474	490	559	
**Non-vegetable nitrate from meat etc. †**	**Q1** **OR (95% CI)**	**Q2** **OR (95% CI)**	**Q3** **OR (95% CI)**	**Q4** **OR (95% CI)**	***p*-value for trend**
Un-adjusted Model	1 [Reference]	1.12 (0.98–1.28)	1.16 (1.06–1.33) ^a^	1.37 (1.20–1.56) ^a^	<0.0001
Model 1	1 [Reference]	1.12 (0.97–1.28)	1.15 (1.00–1.31) ^a^	1.31 (1.15–1.49) ^a^	0.001
Model 2	1 [Reference]	1.10 (0.96–1.26)	1.12 (0.97–1.29)	1.24 (1.04–1.47) ^a^	0.1
Model 3	1 [Reference]	1.12 (0.97–1.28)	1.14 (0.99–1.32)	1.27 (1.07–1.51) ^a^	0.06
**Non-vegetable nitrate from fruit etc.**	**Q1** **(<4.1 mg/day)**	**Q2** **(4.1–6.2 mg/day)**	**Q3** **(6.3–8.6 mg/day)**	**Q4** **(>8.6 mg/day)**	
Number of self-reported CVD related cases	531	512	475	433	
**Non-vegetable nitrate from fruit etc.** **ǂ**	**Q1** **OR (95% CI)**	**Q2** **OR (95% CI)**	**Q3** **OR (95% CI)**	**Q4** **OR (95% CI)**	***p*-value for trend**
Un-adjusted Model	1 [Reference]	0.95 (0.83–1.07)	0.87 (0.76–0.99) ^a^	0.78 (0.68–0.89) ^a^	0.001
Model 1	1 [Reference]	0.96 (0.85–1.10)	0.90 (0.79–1.03)	0.81 (0.71–0.92) ^a^	0.01
Model 2	1 [Reference]	0.98 (0.85–1.12)	0.92 (0.79–1.08)	0.84 (0.68–1.05)	0.4
Model 3	1 [Reference]	0.99 (0.86–1.13)	0.95 (0.81–1.11)	0.88 (0.70–1.09)	0.6

Abbreviations: CVD: Cardiovascular disease; ALSWH: Australian Longitudinal Study on Women’s Health; OR: Odds ratio; 95% CI: 95% confidence intervals. * Total non-vegetable nitrate. Model 1: Adjusted for non-dietary confounders including SES and education. Model 2: Model 1 + dietary confounders including fruit, meat. grains, discretionary choices, processed meat, dairy and alcohol (grams/day). Model 3: Model 2 + energy (kJ/day). a: Indicates OR is statistically significantly (*p* < 0.05) difference from Q1. † Non-vegetable nitrate from meat etc.: included nitrate contributed from meat, discretionary choices, processed meat and alcohol. Model 1: adjusted for non-dietary confounders including SES and education. Model 2: Model 1 + dietary confounders including meat, discretionary choices, processed meat and alcohol (grams/day). Model 3: Model 2 + energy (kJ/day). ǂ Non-vegetable nitrate from fruit etc.: Included nitrate contributed from fruit, grains and dairy foods. Model 1: Adjusted for non-dietary confounders including SES and education. Model 2: Model 1 + dietary confounders including fruit, grains and dairy consumption (grams/day). Model 3: Model 2 + energy (kJ/day).

**Table A2:** Odds of self-reported CVD related complications (sensitivity analysis) by quartile of nitrate intakes in the 1946–1951 cohort of the ALSWH (2001–2016).

Total Nitrate Intakes	Q1 (<45.5 mg/day)	Q2 (45.5–60.2 mg/day)	Q3 (60.3–78.2 mg/day)	Q4 (>78.2 mg/day)	
Number of self-reported CVD related cases	402	411	405	368	
**Total Nitrate Intakes**	**Q1** **OR (95% CI)**	**Q2** **OR (95% C)**	**Q3** **OR (95% CI)**	**Q4** **OR (95% CI)**	***p*-Value for Trend**
Un-adjusted Model	1 [Reference]	1.00 (0.87–1.15)	0.99 (0.86–1.14)	0.90 (0.77–1.04)	0.4
Model 1	1 [Reference]	1.01 (0.87–1.16)	0.97 (0.84–1.12)	0.89 (0.77–1.03)	0.3
Model 2	1 [Reference]	0.97 (0.83–1.12)	0.91 (0.77–1.07)	0.79 (0.64–0.97) ^a^	0.1
Model 3	1 [Reference]	0.96 (0.83–1.12)	0.90 (0.78–1.06)	0.78 (0.63–0.95) ^a^	0.08
**Vegetable Nitrate Intakes**	**Q1** **(<34.8 mg/day)**	**Q2** **(34.8–47.8 mg/day)**	**Q3** **(47.9–64.4 mg/day)**	**Q4** **(>64.4 mg/day)**	
Number of self-reported CVD related cases	421	400	395	370	
**Vegetable Nitrate Intakes**	**Q1** **OR (95% CI)**	**Q2** **OR (95% CI)**	**Q3** **OR (95% CI)**	**Q4** **OR (95% CI)**	***p*-Value for Trend**
Un-adjusted Model	1 [Reference]	0.93 (0.81–1.07)	0.91 (0.79–1.05)	0.86 (0.74–0.99) ^a^	0.2
Model 1	1 [Reference]	0.93 (0.81–1.07)	0.91 (0.79–1.05)	0.85 (0.74–0.99) ^a^	0.2
Model 2	1 [Reference]	0.89 (0.77–1.03)	0.84 (0.72–0.99) ^a^	0.75 (0.61–0.91) ^a^	0.04
Model 3	1 [Reference]	0.89 (0.77–1.03)	0.84 (0.72–0.99) ^a^	0.75 (0.61–0.91) ^a^	0.04
**Total Non-Vegetable Nitrate**	**Q1** **(<8.9 mg/day)**	**Q2** **(8.9–11.7 mg/day)**	**Q3** **(11.8–14.9 mg/day)**	**Q4** **(>14.9 mg/day)**	
Number of self-reported CVD related cases	405	375	393	413	
**Total Non-Vegetable Nitrate Intakes**	**Q1** **OR (95% CI)**	**Q2** **OR (95% CI)**	**Q3** **OR (95% CI)**	**Q4** **OR (95% CI)**	***p*-Value for Trend**
Un-adjusted Model	1 [Reference]	0.91 (0.78–1.05)	0.96 (0.84–1.11)	1.02 (0.89–1.17)	0.4
Model 1	1 [Reference]	0.91 (0.79–1.05)	0.97 (0.84–1.12)	1.00 (0.87–1.15)	0.5
Model 2	1 [Reference]	0.94 (0.81–1.09)	1.02 (0.87–1.20)	1.06 (0.86–1.31)	0.5
Model 3	1 [Reference]	0.94 (0.81–1.10)	1.03 (0.87–1.21)	1.07 (0.87–1.32)	0.5
**Non-Vegetable Nitrate from Meat etc. †**	**Q1** **(<3.6 mg/day)**	**Q2** **(3.6–5.5 mg/day)**	**Q3** **(5.6–8.1 mg/day)**	**Q4** **(>8.1 mg/day)**	
Number of self-reported CVD related cases	345	384	406	451	
**Non-Vegetable Nitrate from Meat etc. †**	**Q1** **OR (95% CI)**	**Q2** **OR (95% CI)**	**Q3** **OR (95% CI)**	**Q4** **OR (95% CI)**	***p*-Value for Trend**
Un-adjusted Model	1 [Reference]	1.12 (0.97–1.30)	1.20 (1.04–1.39) ^a^	1.38 (1.19–1.59) ^a^	0.0001
Model 1	1 [Reference]	1.12 (0.97–1.30)	1.17 (1.01–1.36) ^a^	1.31 (1.13–1.51) ^a^	0.004
Model 2	1 [Reference]	1.10 (0.94–1.28)	1.13 (0.97–1.33)	1.22 (1.10–1.48) ^a^	0.3
Model 3	1 [Reference]	1.12 (0.96–1.30)	1.16 (0.99–1.36)	1.25 (1.03–1.52) ^a^	0.1
**Non-Vegetable Nitrate from Fruit etc.** **ǂ**	**Q1** **(<4.1 mg/day)**	**Q2** **(4.1–6.2 mg/day)**	**Q3** **(6.3–8.6 mg/d** **ay** **)**	**Q4** **(>8.6 mg/day)**	
Number of self-reported CVD related cases	430	425	383	348	
**Non-Vegetable Nitrate from Fruit etc.** **ǂ**	**Q1** **OR (95% CI)**	**Q2** **OR (95% CI)**	**Q3** **OR (95% CI)**	**Q4** **OR (95% CI)**	***p*-Value for Trend**
Un-adjusted Model	1 [Reference]	0.97 (0.85–1.12)	0.87 (0.75–1.00)	0.78 (0.67–0.89) ^a^	0.002
Model 1	1 [Reference]	0.99 (0.86–1.14)	0.90 (0.78–1.04) ^a^	0.81 (0.69–0.93) ^a^	0.01
Model 2	1 [Reference]	1.00 (0.86–1.17)	0.92 (0.77–1.09)	0.83 (0.65–1.06)	0.3
Model 3	1 [Reference]	1.01 (0.87–1.18)	0.94 (0.79–1.13)	0.87 (0.68–1.11)	0.5

Abbreviations: CVD: Cardiovascular disease; ALSWH: Australian Longitudinal Study on Women’s Health; OR: Odds ratio; 95% CI: 95% confidence intervals. CVD related cases for sensitivity analysis: Cases defined as a case if reported >1 time point, unless the case was reported for the first time at Survey 8 (the last survey). Model 1: Adjusted for non-dietary confounders including SES and education. Model 2: Model 1 + major dietary source of nitrate (grams/day). Model 3: Model 2 + energy (kJ/day). a: Indicates OR is statistically significantly (*p* < 0.05) difference from Q1. † Non-vegetable nitrate from meat etc.: Included nitrate contributed from meat, discretionary choices, processed meat and alcohol. ǂ Non-vegetable nitrate from fruit etc.: included nitrate contributed from fruit, grains and dairy foods.

**Table A3:** Odds of self-reported hypertension (sensitivity analysis) by quartile of nitrate intakes in the 1946–1951 cohort of the ALSWH (2001–2016).

Total Nitrate Intakes	Q1 (<45.5 mg/day)	Q2 (45.5–60.2 mg/day)	Q3 (60.3–78.2 mg/day)	Q4 (>78.2 mg/day)	
Number of self-reported hypertension	414	423	404	374	
**Total Nitrate Intakes**	**Q1** **OR (95% CI)**	**Q2** **OR (95% CI)**	**Q3** **OR (95% CI)**	**Q4** **OR (95% CI)**	***p*-Value for Trend**
Un-adjusted Model	1 [Reference]	1.00 (0.87–1.15)	0.95 (0.83–1.10)	0.88 (0.76–1.02)	0.3
Model 1	1 [Reference]	1.01 (0.88–1.16)	0.94 (0.82–1.09)	0.88 (0.77–1.02)	0.2
Model 2	1 [Reference]	0.98 (0.84–1.13)	0.89 (0.75–1.04)	0.79 (0.65–0.97) ^a^	0.09
Model 3	1 [Reference]	0.97 (0.84–1.12)	0.88 (0.74–1.03)	0.78 (0.63–0.95) ^a^	0.06
**Vegetable Nitrate Intakes**	**Q1** **(<34.8 mg/day)**	**Q2** **(34.8–47.8 mg/day)**	**Q3** **(47.9–64.4 mg/day)**	**Q4** **(>64.4 mg/day)**	
Number of self-reported hypertension	430	415	399	371	
**Vegetable Nitrate Intakes**	**Q1** **OR (95% CI)**	**Q2** **OR (95% CI)**	**Q3** **OR (95% CI)**	**Q4** **OR (95% CI)**	***p*-Value for Trend**
Un-adjusted Model	1 [Reference]	0.94 (0.82–1.09)	0.90 (0.78–1.04)	0.84 (0.73–0.97) ^a^	0.1
Model 1	1 [Reference]	0.95 (0.82–1.09)	0.90 (0.78–1.04)	0.84 (0.73–0.97) ^a^	0.1
Model 2	1 [Reference]	0.91 (0.78–1.05)	0.83 (0.71–0.98) ^a^	0.74 (0.61–0.90) ^a^	0.02
Model 3	1 [Reference]	0.91 (0.78–1.05)	0.84 (0.71–0.98) ^a^	0.74 (0.61–0.90) ^a^	0.02

Abbreviations: ALSWH: Australian Longitudinal Study on Women’s Health; OR: Odds ratio; 95% CI: 95% confidence intervals. Model 1: Adjusted for non-dietary confounders including SES and education. Model 2: Model 1 + major dietary source of nitrate (grams/day). Model 3: Model 2 + energy (kJ/day). a: Indicates OR is statistically significantly (*p* < 0.05) difference from Q1.
