# Supplementary material for: Vegetable Nitrate Intakes Are Associated with Reduced Self-Reported Cardiovascular-Related Complications within a Representative Sample of Middle-Aged Australian Women, Prospectively Followed up for 15 Years
Source: Nutrients. 2019 Jan 22;11(2):240. doi: 10.3390/nu11020240 (PMC6412377; doi:10.3390/nu11020240)
Supplement: Supplementary file 1 [file nutrients-11-00240-s001.pdf]

Supplementary Materials

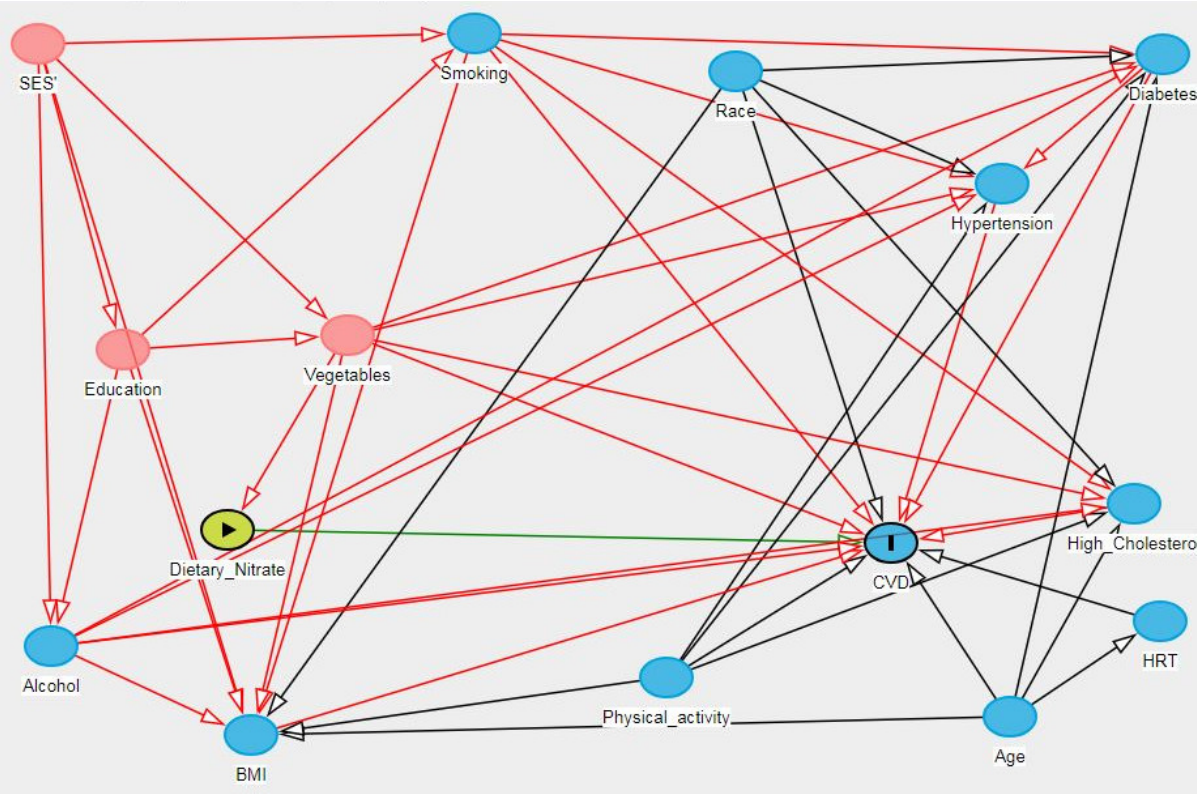

Figure S1. Directed Acyclic Graph (DAG). Used to identify confounders between dietary nitrate and self-reported cardiovascular diseases.
